# Evaluation in broilers of aerosolized nanoparticles vaccine encapsulating imuno-stimulant and antigens of avian influenza virus/*Mycoplasma gallisepticum*

**DOI:** 10.1186/s12917-020-02539-5

**Published:** 2020-08-31

**Authors:** Taha Kumosani, Soonham Yaghmoor, Wesam H. Abdulaal, Elie Barbour

**Affiliations:** 1 Department of Biochemistry, Faculty of Science, Experimental Biochemistry Unit, King Fahd Medical Research Center and Production of Bioproducts for Industrial Applications Research Group, King Abdulaziz University (KAU), Jeddah, Kingdom of Saudi Arabia; 2 Experimental Biochemistry Unit, King Fahd Medical Research Center and Production of Bioproducts for Industrial Applications Research Group, KAU, Jeddah, Kingdom of Saudi Arabia; 3 Department of Biochemistry, Faculty of Science, King Abdulaziz University, Jeddah, Kingdom of Saudi Arabia; 4 Adjunct to Department of Biochemistry, Faculty of Science, KAU, Jeddah, Kingdom of Saudi Arabia; 5 Director of R and D Department, Opticon Hygiene Consulting, Oechsli 7, 8807 Freienbach, Switzerland

**Keywords:** Aerosolized vaccine, Broilers, Nanoparticles, Antigens, *Echinacea*, H9N2, MG

## Abstract

**Background:**

The global prevalence of economic primary infection of poultry by H9N2 virus, including the Lineage A, panzootic group ME1, and associated with secondary infection by *Mycoplasma gallisepticum* (MG), is alarming to the sustainability of the poultry sector. This research evaluated in broilers the immunity and protection induced by aerosolization of liposomal nanoparticles vaccine, encapsulating antigens of H9N2 virus and MG, with or without the incorporation of *Echinacea* extract (EE) immuno-stimulant. Six different treatments (TRTs) of broilers were included in the experimental design, with three replicate pens/TRT and stocking of 20 day-old birds/replicate.

**Results:**

The tracheobronchial washings of birds subjected to aerosolization of liposomal nanoparticles, encapsulating antigens of H9N2 and MG and EE had the highest significant mean levels of each of IgA and IgG specific to H9N2 and MG, associated with lowest tracheal MG colonization, tracheal H9N2 recovery, tracheal histopathologic lesions, mortality, and best performance in body weight and feed conversion compared to all other challenged birds allocated to different treatments (*P* < 0.05). However, the control broilers, free from challenge with MG and H9N2, had the lowest mortality and tracheal lesions, and the highest production performance.

**Conclusion:**

The aerosolization of liposomal nanoparticles, encapsulating antigens of H9N2 and MG and EE resulted in enough local immunity for protection of broilers against infection, and in attaining the highest production performance in challenged birds. The potential implication of vaccinating with safe killed nanoparticle vaccines is of utmost importance to the global poultry sector.

## Background

The H9N2 subtype is the most prevalent Low Pathogenic Avian Influenza Virus (LPAIV) in poultry sector of the world [1–4]. All Middle East and North African (MENA) countries reported an endemicity of this subtype within Lineage A viruses, which originated from Pakistan in the year 1998 [4], included also in the panzootic group ME1, recovered from poultry outbreaks in Israel, Kingdom of Saudi Arabia (KSA), Libya, Morocco, Qatar, Tunisia, and UAE [4].

The coinfection of H9N2 with *Mycoplasma gallisepticum* (MG) augmented significantly their individual pathogenicity in poultry, leading into significant losses in production [5–8]. The prevalence of MG is also global [9], including its prevalence in poultry of the MENA region [10, 11]. In addition, the impact of H9N2 on human health is reported recently, in which an emergence of a novel H7N9 reassortant subtype was found to cause severe human respiratory infections in China [12]. Bioinformatic analyses of the H7N9 virus revealed an acquisition of its six internal genes from H9N2 avian influenza viruses of chickens [13].

The current control of H9N2 and MG infection in poultry is mainly by administering killed injectable vaccines [14, 15], an approach that doesn’t provide enough local immunity in the respiratory system of the host, resulting in persistence of infection and incomplete protection [14, 16]. The constraints related to application of live vaccines for protection against Avian Influenza (AI) impacted the global prevalence in poultry control programs of commercial inactivated AI vaccines (95.5%) over the live ones (4.5%) [17]. Similarly, the control of MG in poultry is favored by killed MG vaccines, in avoidance of epithelial injuries that could result from live strains [18–20].

Scientists are currently searching for alternatives to the costly injectable killed vaccines, and to the injurious approach of administering live vaccine strains of H9N2 and MG in poultry. Preliminary attempts in deliverance of soluble antigens through the nasal passage, for immunization against AI [21, 22] and MG [23], are documented. Such attempts led to sporadic acquirement of immune responses [21–23], with a need to augment the induction of local immunity in the respiratory tract, and to establish a relationship between this augmentation and protection against coinfection by MG and H9N2.

It is worth noting that phospholipids constitute 75% of the protective epithelial layer of the respiratory tract [24, 25]; thus, the nasal administration of phospholipids in the form of liposomal nanoparticles, is believed to replace the lost part of cell membranes that are caused by microbial injury [26–29]. Actually, nasal administration of liposomes alone, without encapsulating any medicine in it, is commercialized in Germany for human respiratory tract therapy since the year 2007 [30].

The search for immuno-stimulants targeting the epithelial layer of the respiratory system in poultry and its impact on protection against avian influenza alone or in coinfection with MG, are not given enough attention during the last decades [21, 31–34]. The first work on evaluation of liposomal vaccines in poultry, administered via the subcutaneous route, and constructed of cationic micelles, is documented in the year 1987 [35]. In addition, the pioneer determination of the *Echinacea* dosing in poultry, was able to induce significant immuno-stimulation to specific antigens in the administered vaccines [36], a dosing that was successfully extrapolated to ewes, which led to significant seroconversion to another administered antigens [37].

Based on the above literature, a hypothesis is established, aiming at an aerosolization of soluble antigens of H9N2 and MG microorganisms and *Echinacea* Extract (EE), encapsulated within cationic liposomal nanoparticles, predicting that it could augment the local immunity in the respiratory system of the broilers, which might improve protection against infection, histopathologic lesions, and mortality induced by a challenge with viable H9N2 and MG.

## Results

### Local immunity

The completely randomized experimental design, implemented in this research and presented in Table 1, helped in performing the ANOVA for comparison of means of local immunity parameters (Table 2). The assessed parameters included the H9N2-specific IgA, H9N2-specific IgG, MG-specific IgA, and MG-specific IgG.

**Table 1:** Experimental Design of differently treated broilers

Treatment^a^	Aerosolization by liposomal nanoparticles encapsulating the below materials^b^		Challenge^c^
	Solubilized antigens of H9N2 and MG	*Echinacea* extract (EE)	
1	+	–	+
2	–	+	+
3	+	+	+
4	–	–	+
5	+	+	–
6	–	–	–

^a^Each treatment had three replicate pens, with 20 birds/pen

^b^Aerosolization of 2 ml/bird at a distance of 20 cm from nostrils, and at the age of 14 and 21 d

^c^Bivalent intra-tracheal challenge at 28 days of age with 0.5 ml/bird of each of H9N2 and MG, at individual concentration of 4 HA units/50 μl

**Table 2:** Impact of aerosolization of liposomal chicken vaccine^a^, incorporating H9N2 and MG antigens in presence and absence of *Echinacea* extract (EE), on mucosal immunity to H9N2

Treatment^b^	Mean ELISA – O.D. values of IgA/IgG specific to H9N2 at different ages (d)			
	14	21	28^c^	35
1	0.0^1^/0.0^1^	0.2^1^/0.6^1^	0.5^1^/1.2^1^	0.8^1^/1.8^1^
2	0.0^1^/0.0^1^	0.0^2^/0.0^2^	0.0^2^/0.0^2^	0.3^2^/0.9^2^
3	0.0^1^/0.0^1^	0.6^3^/1.5^3^	0.9^3^/2.3^3^	1.5^3^/2.9^3^
4	0.0^1^/0.0^1^	0.0^1^/0.0^2^	0.0^1^/0.0^1^	0.1^4^/0.4^4^
5	0.0^1^/0.0^1^	0.5^3^/1.6^3^	0.7^3^/2.2^3^	1.0^5^/2.1^5^
6	0.0^1^/0.0^1^	0.0^2^/0.0^2^	0.0^2^/0.0^2^	0.0^4^/0.0^6^

^a^Vaccination and/or EE administration to broilers at 14 and 21 days of age

^b^TRT 1 = Vaccine administration containing H9N2 and MG antigens and challenged; TRT 2 = EE administration only and challenged; TRT 3 = Vaccine administration containing H9N2, MG, EE, and challenged; TRT 4 = Only challenged (positive controls); TRT 5 = Vaccine administration containing H9N2, MG, EE, and deprived of challenge; TRT 6 = Deprived of antigens and EE, and of challenge (Negative Controls)

^c^Challenge at 28 days of age with 4HA units/50 μl of each of H9N2 and MG

^1-6^Means in numerators of a column followed by different Arabic numerical - superscripts are significantly different (*P* < 0.05); similarly, means in denominators of a column followed by different Arabic - numerical superscripts are also significantly different (*P* < 0.05)

#### H9N2-specific IgA

The mean IgA level, specific to aerosolized H9N2 antigens that were incorporated in nanoparticles, and determined in broilers’ tracheobronchial washings at 14, 21, 28, and 35 d of age, showed its early detection at 21 d of age, only in broilers of TRTs 1, 3, and 5 that were vaccinated at d 14. There was a significantly higher mean H9N2 specific-IgA in birds of TRTs 3 and 5 that received the EE-supplemented vaccine, compared to that in birds of TRT 1 that were deprived of this supplementation (*P* < 0.05). The boosting by the aerosolized vaccine at 21 d of age in broilers of TRTs 1, 3, and 5, raised their H9N2-specific IgA titers at 28 d of age, with significantly higher titers in birds of TRTs 3 and 5 compared to that of TRT 1 (P < 0.05). The bivalent aerosolized challenge by homologous viable H9N2 and MG microorganisms at 28 d of age in birds of TRTs 1–4 resulted in highest significant H9N2-specific IgA titer in birds of TRT 3 that were administered the EE-supplemented vaccine compared to all other birds (*P* < 0.05). The challenged birds in TRT 4, that were deprived of vaccination and EE (Positive Controls), had significantly lowest H9N2-specific IgA titer at 35 d of age compared to all other challenged birds (P < 0.05), while the negative controls in TRT 6 kept an undetectable titer at different ages.

#### H9N2-specific IgG

The mean H9N2-specific IgG, detected in tracheobronchial washings of the six differently treated broilers, are shown in (Table 2). The dynamic pattern obtained by H9N2-specific IgG titers was similar to that obtained by the H9N2-specific IgA isotype, with an apparent difference in quantities, in which the mean titers of the IgG isotype were always higher than that of the IgA, and at different ages.

#### MG-specific IgA

The mean MG-specific IgA in the tracheobronchial washings of the six differently treated broilers is shown in (Table 3). The detection of early MG-specific IgA at 21 d of age (one week post priming with the bivalent vaccine) was present only in birds of TRT 3; however, the response was not significantly different from that of the other treatments (*P* > 0.05). The boosting by the aerosolized bivalent vaccine at 21 d of age created detectable means of MG-specific IgA titer in vaccinated birds of TRTs 1, 3, and 5 at 28 d of age; in addition, birds of TRTs 3 and 5, vaccinated and EE immuno-stimulated, had significantly higher mean MG-specific IgA titer compared to that in birds of TRT 1 that were administered the antigens in the vaccine, but devoid of the EE immuno-stimulant (*P* < 0.05). The bivalent challenge with H9N2 and MG at 28 d of age in birds of TRTs 1–4 resulted after one week in highest significant MG-specific IgA in birds of TRT 3, that were vaccinated and immuno-stimulated by EE. It is worth noting that the administration of antigens devoid of EE in birds of TRT 1, or administration of EE alone to birds of TRT 2 resulted in significantly lower mean MG-specific IgA titers post one week of challenge compared to response in challenged birds of TRT 3 that were administered the vaccine incorporating both the antigens and EE (*P* < 0.05).

**Table 3:** Impact of aerosolization of liposomal chicken vaccine^a^, incorporating H9N2 and MG antigens in presence and absence of *Echinacea* extract (EE), on mucosal immunity to MG

Treatment^b^	Mean ELISA – O.D. values of IgA/IgG specific to MG at different ages (d)			
	14	21	28^c^	35
1	0.0^1^/0.0^1^	0.0^1^/0.4^1^	0.4^1^/0.9^1^	0.6^1^/1.4^1^
2	0.0^1^/0.0^1^	0.0^1^/0.0^2^	0.0^2^/0.0^2^	0.3^2^/0.7^2^
3	0.0^1^/0.0^1^	0.2^1^/0.9^3^	0.7^3^/1.8^3^	1.2^3^/2.4^3^
4	0.0^1^/0.0^1^	0.0^1^/0.0^2^	0.0^1^/0.0^1^	0.0^4^/0.2^4^
5	0.0^1^/0.0^1^	0.0^1^/0.8^3^	0.6^3^/1.9^3^	0.8^5^/1.8^5^
6	0.0^1^/0.0^1^	0.0^1^/0.0^2^	0.0^2^/0.0^2^	0.0^6^/0.0^6^

^a^Vaccination and/or EE administration to broilers at 14 and 21 days of age

^b^TRT 1 = Vaccine administration containing H9N2 and MG antigens and challenged; TRT 2 = EE administration only and challenged; TRT 3 = Vaccine administration containing H9N2, MG, EE, and challenged; TRT 4 = Only challenged (positive controls); TRT 5 = Vaccine administration containing H9N2, MG, EE, and deprived of challenge; TRT 6 = Deprived of antigens and EE, and of challenge (Negative Controls)

^c^Challenge at 28 days of age with 4HA units/50 μl of each of H9N2 and MG

^1-6^Means in numerators of a column followed by different Arabic numerical -superscripts are significantly different (P < 0.05); similarly, means in denominators of a column followed by different Arabic numerical - superscripts are also significantly different (P < 0.05)

#### MG-specific IgG

The mean MG specific-IgG in the tracheobronchial washings of the six differently treated broilers are also shown in (Table 3). The pattern of early IgG response at one week post priming by MG antigens was different than that obtained for MG-specific IgA; detectable MG specific-IgG titers were shown in birds of TRTs 1, 3, and 5 at one week post priming, while the MG specific-IgA titers post one week of priming were only detected in birds of TRT 3. The MG specific-IgG response pattern at 28 and 35 days of age (Table 3) was similar to that obtained for H9N2 specific-IgG response (Table 2), in which the highest significant MG specific-IgG response at these two ages was obtained by birds of TRT 3 that were administered the vaccine incorporating both the antigens and EE (*P* < 0.05); however, the lowest response to challenge was obtained by birds of TRT 4 that were unvaccinated and deprived of the immuno-stimulant (*P* < 0.05).

### Protection against tracheal colonization by MG and infection by H9N2

Table 4 showed the impact of the different treatments of broilers on tracheal colonization by MG and infection by H9N2 at 35 d of age. The lowest recovered colonies of MG per tracheal swab at 35 d of age, among challenged birds of the four TRTs, were that in birds of TRT 3 (*P* < 0.05), associated with the highest IgA and IgG specific to MG in their tracheobronchial washings that were collected at the challenge age of 28 d (Table 3). In addition, the mean recovery of tracheal H9N2 was the lowest in birds of TRT 3 compared to that of all challenged birds (*P* < 0.05); this low recovery of H9N2 in birds of TRT 3 was associated also with the highest means of H9N2-specific IgA and IgG at challenge age of 28 d, compared to means of the two isotypes in challenged birds of the other TRTs (P < 0.05) (Table 2). The recovery data of MG and H9N2 in challenged birds showed the highest protection in birds of TRT 3 that were aerosolized by the vaccine that incorporated both the antigens and EE, compared to birds administered only the antigens (TRT 1), or only the EE (TRT 2), or birds deprived of both the antigens and the EE (TRT 4). The immuno-modulation acquired by the antigens and EE (Tables 2 and 3) had a positive effect on protection against infection by MG and H9N2 (Table 4). It is worth noting that birds in the unchallenged TRTs 5 and 6 were devoid of tracheal MG colonization and H9N2 infection by the market age of 35 days.

**Table 4:** Impact of aerosolization of liposomal chicken vaccine^a^, incorporating H9N2 and MG antigens in presence and absence of *Echinacea* extract (EE), on protection in challenged^b^ broilers against respective colonization and infection by MG and H9N2

Treatment^c^	Mean *Mycoplasma gallisepticum* colony count At 35 d of age (CFU/tracheal swab)	Mean recovery % Of tracheal H9N2 at 35 d of age
1	(2.5 × 10^2^)^1^	(27.0)^1^
2	(5.0 × 10^2^)^2^	(42.0)^2^
3	(3.0 × 10)^3^	(3.0)^3^
4	(2.0 × 10^4^)^4^	(86.0)^4^
5	(0.0)^5^	(0.0)^5^
6	(0.0)^5^	(0.0)^5^

^a^Vaccination and/or EE administration to broilers at 14 and 21 days of age

^b^Challenge at 28 days of age with 4HA units/50 μl of each of H9N2 and MG

^c^TRT 1 = Vaccine administration containing H9N2 and MG antigens and challenged; TRT 2 = EE administration only and challenged; TRT 3 = Vaccine administration containing H9N2, MG, EE, and challenged; TRT 4 = Only challenged (positive controls); TRT 5 = Vaccine administration containing H9N2, MG, EE, and deprived of challenge; TRT 6 = Deprived of antigens and EE, and of challenge (Negative Controls)

^1-5^Bracketed means in a column followed by different Arabic numerical - superscripts are significantly different (P < 0.05)

### Protection against tracheal histopathologic lesions and mortality

Another data related to protection against the controlled bivalent challenge at 28 days of age is presented in Table 5, showing the impact of the six different TRTs on frequency of each of three tracheal histopathologic lesions at 35 d of age, and on cumulative mortality %. The lowest frequency of the three tracheal lesions namely, deciliation, hypertrophy, and goblet cells degeneration, and the lowest cumulative mortality % were in birds of TRT 3 compared to challenged birds of the other three TRTs (*P* < 0.05). The unchallenged birds of TRTs 5 and 6 had the lowest mortality and almost an absence of the three tracheal lesions compared to all challenged birds.

**Table 5:** Impact of aerosolization of liposomal chicken vaccine^a^, incorporating H9N2 and MG antigens in presence and absence of *Echinacea* extract (EE), on protection of challenged^b^ broilers against tracheal histopathologic lesions and mortality

Treatment^c^	Mean frequency of tracheal histopathologic lesions and mortality at 35 d of age			
	% Trachea with histopathogic lesions			Cumulative mortality %
	Deciliation	Mucosal hypertrophy	Goblet cells degeneration	
1	51.0^1^	58.0^1^	19.0^1^	22.0^1^
2	62.0^2^	68.0^2^	27.0^2^	35.0^2^
3	5.5^3^	7.0^3^	2.5^3^	4.0^3^
4	72.0^4^	85.0^4^	32.0^4^	45.0^4^
5	4.7^3^	6.5^3^	1.8^5^	2.0^5^
6	3.1^5^	4.2^5^	1.0^5^	2.0^5^

^a^Vaccination and/or EE administration to broilers at 14 and 21 days of age

^b^Challenge at 28 days of age with 4HA units/50 μl of each of H9N2 and MG

^c^TRT 1 = Vaccine administration containing H9N2 and MG antigens and challenged; TRT 2 = EE administration only and challenged; TRT 3 = Vaccine administration containing H9N2, MG, EE, and challenged; TRT 4 = Only challenged (positive controls); TRT 5 = Vaccine administration containing H9N2, MG, EE, and deprived of challenge; TRT 6 = Deprived of antigens and EE, and of challenge (Negative Controls)

^1-5^Means in a column followed by different Arabic numerical - superscripts are significantly different (P < 0.05)

### Production performance

Table 6 presented the production data of broilers in the six treatments. The highest significant body weight and lowest FCR at market age was observed in birds of TRT 3 compared to that in challenged birds of TRTs 1, 2, and 4. (*P* < 0.05). However, the unchallenged birds of TRTs 5 and 6 had the highest growth and lowest feed conversion compared to all challenged birds.

**Table 6:** Performance of the differently treated broilers at the market age of 35 days

Treatment^a^	Live body weight (g)	Feed Conversion Ratio
1	2100^1^	1.62^1^
2	2042^2^	1.72^2^
3	2150^3^	1.57^3^
4	1920^4^	1.85^4^
5	2198^5^	1.50^5^
6	2205^5^	1.54^5^

^a^TRT 1 = Vaccine administration containing H9N2 and MG antigens and challenged; TRT 2 = EE administration only and challenged; TRT 3 = Vaccine administration containing H9N2, MG, EE, and challenged; TRT 4 = Only challenged (positive controls); TRT 5 = Vaccine administration containing H9N2, MG, EE, and deprived of challenge; TRT 6 = Deprived of antigens and EE, and of challenge (Negative Controls)

^1-5^Means in a column followed by different Arabic numerical - superscripts are significantly different (P < 0.05)

## Discussion

### Local immunity

To our knowledge, this is the first observation of an early detection of IgA specific to H9N2 in tracheobronchial washings of 21 d old broilers that were administered the aerosolized nanoparticle vaccine at 14 d of age (Trts 1, 3 and 5); this vaccination resulted in significantly higher mean H9N2-specific IgA level in birds of TRTs 3 and 5 that received the EE-supplemented vaccine, compared to birds of TRT 1 that were deprived of this supplementation (P < 0.05). No documentations are present in literature, showing the significant immuno-stimulation by EE, as that detected in birds of TRTs 3 and 5. It is worth noting that *Echinacea* immune-stimulations to other antigens were reported previously in avians and ruminants [36, 37]; most likely, the alkamides of EE were involved in the immunomodulatory properties, leading to IgA expression [38, 39], while the other active ingredient of EE namely, the polysaccharides, could have provided the anti-inflammatory effect [40]. The administration of the booster vaccine to broilers of TRTs 1, 3, and 5, raised their IgA titers at 28 d of age, while maintaining significantly higher titers in birds of TRTs 3 and 5 compared to that of TRT 1 (*P* < 0.05). It is documented that vaccine boosting in chickens by mucosal killed vaccines led to significant conversion in local antibodies [41]. The following bivalent aerosolized challenge by homologous viable H9N2 and MG microorganisms at 28 d of age in birds of TRTs 1–4 resulted in highest significant H9N2-specific IgA titer in birds of TRT 3 that were administered the EE-supplemented vaccine compared to all other birds (P < 0.05). This is indicative of the EE - efficient role in priming the immune system before the challenge occurs [42], a result that is in agreement with previous reports, showing the benefits of other immunopotentiators on the local immunity to intra-nasally administered killed vaccines [43, 44]; actually, the positive and negative controls, deprived of priming with the vaccine had lower H9N2 – specific IgA compared to primed birds; an agreeable observation with that of previous documentations [43, 44].

The dynamic pattern obtained by IgG titers in tracheobronchial washings was similar to that obtained by the IgA isotype, with an apparent difference in quantified amounts; actually, the mean titers of the IgG isotype were always higher than that of the IgA, and at different ages (Table 2). It is reported that the IgG-containing cells are prevalent in mucosal tissue, especially in the nasal cavity [45]. This induction by nasally administered antigens of higher local IgG level in the respiratory tract of chicken compared to IgA is in agreement with previous documented result [46]. It is worth noting that the IgA producing - plasma cells are prevalent in Harderian gland, supplying this isotype to limited segment of the upper respiratory system of chicken [47].

The insignificant IgA response to MG antigens at one week post priming (Table 3) in comparison to the significant IgA responses to H9N2 antigens, detected in birds of TRTs 1, 3, and 5 (Table 2), is most likely due to weaker immunogenicity of MG antigens compared to that of H9N2; previous researches pointed at the weak immunogenicity nature of MG antigens [48]; however, the boosting by the aerosolized bivalent vaccine at 21 d of age was able to induce detectable MG-specific IgA titers in vaccinated birds of TRTs 1, 3, and 5 at 28 d of age; however, higher mean titers were detected in vaccinated and EE immuno-stimulated birds of TRTs 3 and 5 compared to birds of TRT 1 that were administered the same antigens but devoid of the EE immuno-stimulant (*P* < 0.05). The administration of vaccine booster, incorporating the immuno-stimulant EE, to birds of TRTs 3 and 5 seems indispensable for obtaining a significantly higher local MG-specific IgA compared to that of birds in TRT 1, a data that is in agreement with previous reports [23]. Results demonstrated in Table 3 emphasized the need of two aerosolized liposomal vaccinations and the incorporation of EE (TRT 3), before the challenge at 28 d of age, in order to obtain the highest mean MG-specific IgA compared to other treatments; Previous documentations showed that an administration of a booster of killed vaccine containing MG antigens, in association with a supplementation of immuno-stimulant, will elevate the local MG specific-IgA in chicken [23].

The detection of MG specific - IgG titers in birds of TRTs 1, 3, and 5 at one week post priming (Table 3) was noticeable, while the detection of MG specific-IgA titers post one week of priming was restricted only to birds of TRT 3; this is most likely due to the presence of abundant IgG - producing B-cell clones in the respiratory system of birds compared to scarcity of secretory IgA - producing B-cells [45]. The highest significant MG specific-IgG response post challenge was obtained by birds of TRT 3 that were administered the vaccine incorporating both the antigens and EE, providing another evidence related to immune-stimulation by the *Echinacea* Extract.

### Protection against respective tracheal colonization and infection by MG and H9N2

The lowest recovery of tracheal MG and H9N2 from challenged birds was in those allocated to TRT 3 (Table 4), who had the appropriate priming that led to the highest MG and H9N2 – specific IgA and IgG in their tracheobronchial washings at the challenge age of 28 d (Table 3). Previous reports documented the role of IgA and IgG in local protection against tracheal infection of broilers by the two organisms [21–23, 49, 50], emphasizing the role of mucosal immunity in projects targeting the control of these two pathogens in poultry.

The immuno-modulation by delivering both the antigens and EE (Tables 2 and 3) had a positive effect on protection against MG and H9N2 (Table 4). Previous works demonstrated the role of EE in immuno-potentiation of chicken against Infectious Bursal Disease Virus (IBDV), *Salmonella* spp., and coccidial spp. [36, 51].

### Protection against histopathologic lesions and mortality

There was an apparent impact of acquired high local immunity of IgA and IgG (Tables 2 and 3) on protection against tracheal injuries and mortality by the bivalent challenge, as demonstrated by birds of TRT 3 (Table 5). Previous works included images of tracheal histopathologic lesions created by H9N2, MG, and by coinfection with both organisms [52]; the protection by IgA and IgG against histopathologic lesions, induced individually by MG or H9N2 in broilers, are previously documented [42, 53]. It is worth noting that the unchallenged birds of TRTs 5 and 6 had the lowest mortality and frequency of the three tracheal lesions compared to all challenged birds. This shows the importance of rearing broilers without exposure to MG and H9N2, an approach that is managed solely by efficient biosecurity [54] and by rearing day-old birds that are offsprings of MG free -breeders [55].

### Production performance

The obtained lowest tracheal injuries in challenged birds of TRT 3 compared to the frequency of lesions in the other challenged birds (Table 5) is most likely behind the improvement in performance of birds in TRT 3 (Table 6); this observation is in agreement with similar documented relationship between lesion frequency and performance [56, 57]. However, the unchallenged birds of TRTs 5 and 6 had the highest growth and lowest feed conversion compared to all challenged birds, a data that is in agreement with another published data [58, 59]; the obtained production data emphasize the recommendation of raising birds that are free of infection by the H9N2 and MG.

## Conclusions

The challenged birds of TRT 3 that were primed with the aerosolized liposomal vaccine, incorporating both the antigens of MG and H9N2, and the EE immuno-stimulant, showed the highest local immune responses, protection against tracheal histopathologic lesions, and better production compared to all other challenged birds that either received the liposomal vaccine incorporating only the antigens, or incorporating solely the EE, or deprived totally of vaccination. The obtained data also showed that it is preferable to raise broilers free from any exposure to MG and H9N2, as shown in birds of TRTs 5 and 6, that had the lowest lesion frequency and mortality, associated with the highest live body weight and lowest FCR at the market age.

## Methods

The experimental approach included the origin of the H9N2 and MG strains and the EE extract, the H9N2 and MG solubilization techniques, the preparation protocols in synthesis of three forms of cationic liposomal nanoparticle vaccine, the experimental design in evaluation of three forms of the vaccine in broilers, the immunity and protection of differently treated broilers, and production performance of the birds.

### H9N2/MG strains and Echinacea extract (EE)

The H9N2 strain used in challenge, and in solubilization of its proteins for ELISA preparation and for encapsulation within the liposomal nanoparticles vaccine, is of Lineage A, panzootic group ME1, with typical genome to that reported in Israel, KSA, Libya, Morocco, Qatar, Tunisia, and UAE [4]; this strain is of the same lineage reported by all countries of the Middle East and North Africa [4]. The MG strain, used for the same purpose as that of H9N2, belonged to the virulent R-strain [58, 60]. The EE is alcohol and sugar free liquid (Swanson premium, Fargo, ND, USA), diluted 1:100 with PBS, providing 5.0 mg of each of *E. angustifolia* and *E. purpurea* per milliter of the liposomal vaccine.

### H9N2 and MG solubilization

The solubilization of the proteins contained in a suspension of 4 HA units/0.5 ml of each of H9N2 and MG was accomplished with 1% sodium dodecyl sulfate (SDS) detergent, keeping a ratio of SDS/protein equivalent to 1. This solubilization was completed by continuous shaking at 37^o^ C for 90 min. The solubilized proteins of H9N2 and MG were used to coat the ELISA plates and for its incorporation within the cationic liposomal nanoparticles to produce the adjuvanted killed vaccine**.**

### Preparation of cationic liposomal nanoparticle vaccine

The cationic liposomal nanoparticles vaccine was prepared in human medicine research, as pioneered in the year 1965 [61], and its earliest introduction by us into poultry vaccine research [35]. Briefly, a molar ratio of phosphatidyl choline to cholesterol equivalent to 7:3, and a total lipid concentration of 11.0–13.0 mg/ml were adopted in the synthesis. Stearyl amine was added to create the cationic charged micelles. The detergent used in solubilization of the lipids was sodium cholate, with a molar ratio of phosphatidyl choline: sodium cholate equivalent to 0.5, followed by dialysis against phosphate-buffered saline through a membrane of 10,000 cut-point. The solubilized lipids were dried onto the internal wall of round flask attached to a rotary evaporator. The encapsulation within the micelles of the cationic liposomal nanoparticles was prepared in three different forms, by adding the following to the dried lipid film: Form 1 - Addition of 5.0 μg/μl of each of the solubilized antigens of H9N2 and MG alone; Form 2 - Addition of EE alone (5.0 μg/μl of each of *E. angustofolia,* and *E. purpurea*); Form 3 - Addition of 5.0 μg/μl of each of the solubilized antigens of H9N2 and MG and the EE, in a similar concentration to that shown under Form 1 for antigens and Form 2 for EE. The formed micelles were sonicated for 15 min with a continuous pulse, a 50% duty cycle, and at a 20 KHz untrasonic vibration, forming micelles with an average diameter of 32 μm.

### Experimental design in evaluation of three forms of the vaccine

Table 1 summarizes the completely randomized experimental design implemented in this work, comprised of six treatments, with three replicate pens per treatment (TRT), in which each pen contained 20 day-old meat type chicks, totaling into 360 experimental birds. The day-old birds were obtained from our institution’s hatchery, with a mean weight of 38 g, and weight range of 36–39 g. The inclusion of this number of birds is indispensable for providing unbiased data that will allow the implementation of proper statistical analysis for comparison of assessed parameter means among the six different treatments. The day-old birds were randomly distributed in their pens, reared on the floor that is previously disinfected and covered with wood shaving; the birds were provided with continuous lighting, controlled temperature and feed formulation, following the instructions of Aviagen Co for Ross 308 broiler management. The birds had a continuous access to feeders and drinkers, while abiding by a welfare-related assessment to the broilers environment throughout the whole trial. The specie of the experimental birds was *Gallus Gallus domesticus*, Ross 308 strain, with ratio of males to females equivalent to 1:1. Each of the three forms of the vaccine was aerosolized onto birds of its respective treatment, at a volume of 2 ml/bird, and at a set distance of 20 cm from its nostrils. Birds in TRT 1 were aerosolized, at 14 and 21 days of age, with Form 1 - vaccine and challenged intra-tracheally at 28 days of age with 0.5 ml/bird of each of H9N2 and MG, at individual concentration of 4 HA units/50 μl; Birds of TRT 2 were aerosolized at the same two ages with Form 2 – vaccine and challenged similarly; birds of TRT 3 were aerosolized at same ages with Form 3 - vaccine and challenged similarly; Birds in TRT 4 were deprived of aerosolization and challenged similarly (Positive Controls); Birds in TRT 5 were aerosolized with Form 3 – vaccine at same ages as birds in TRTs 1–3, and kept without challenge; Birds in TRT 6 were deprived of both the aerosolization and challenge (Negative Controls). The experiment was terminated at the market age of 35 days by euthanization, using an injection via the pectoral muscle, equivalent to 20 mg/Kg of tiletamine/zolazepam.

### Immunity and protection of differently treated broilers

#### Local immunity

The ELISA preparation for quantitating the IgA and IgG specific antibodies in the tracheobronchial washings of broilers was adopted according to previous documentations [62, 63]. Briefly, the propagated H9N2 in chicken embryos [64] and MG growth in Frey’s medium [65] were purified and each diluted in PBS to a concentration of 1 mg protein/ml. A volume of 9.0 ml of each was added over 0.9 ml of 1% Sodium dodecyl sulfate (SDS) detergent, keeping a ratio of SDS/protein = 1. The solubilization of each of H9N2 and MG was completed by continuous shaking at 37^o^ C for 90 min.

The microtiter plates for individual detection of IgA specific to H9N2 and that specific to MG were Immunolon 1, coated with 0.18 μg of solubilized proteins of the specific strain/well. The tracheobronchial washings were collected according to our previous documentation [63], and each washing was applied in 50 μl/well at a dilution of 1:5. The biotin labelled anti-chicken IgA conjugate [63] was applied at 1:100 dilution in PBS-tween, while the application of the horseradish peroxidase-labelled avidin, Type IV (Millipore Sigma, Darmstadt, Germany) was diluted to 1:625 in PBS-Tween associated with a supplementation of 1% BSA. The added substrate was 0.034% W/V of o-phenylenediamine (Millipore Sigma, Darmstadt, Germany) and 0.040% (V/V) of 30% H_2_O_2_ in 0.1 M Phosphate-citrate buffer of pH 5.0. The absorbance was read at 492 nm.

The same above procedure was used to quantitate the IgG in tracheobronchial washings that is specific to H9N2 and that for MG antigens, except that the conjugate was a biotin labelled anti-chicken IgG (Novus Biological, CO, USA).

#### Protection against tracheal colonization by MG

Tracheal swabs were collected from birds of the six treatments at 35 days of age, i.e., one week post the bivalent challenge with H9N2 and MG. Each swab was washed in 5 ml of Frey’s medium [58, 65]. The enumeration of the tracheal MG was accomplished by serial dilution of the tracheal swab’s washing, with a dilution factor of 1:10, and plating of 0.1 ml of each dilution in triplicate onto three individual plates of Frey’s agar medium. Plates were incubated for 7 days at 37^o^ C, followed by microscopic counting of the formed colonies with typical fried egg-morphology. The colony forming units was calculated per the original washing of 5 ml, representing the suspended MG cells from the washed swab.

#### Protection against tracheal infection by H9N2

Another collected swabs from birds of the six treatments at 35 days of age, were each washed in 2 ml of transport medium constituted of ratio of glycerol to Phosphate buffered saline equivalent to 1:1. This mix of one liter was supplemented with the following amounts of antimicrobials: benzylpenicillin (2 × 10^6^ IU), streptomycin (200 mg), polymyxin B (2 × 10^6^ IU), gentamicin (250 mg), nystatin (0.5 × 10^6^ IU), ofloxacin hydrochloride (60 mg), and sulfamethoxazole (0.2 g). A volume of 0.1 ml of the tracheal swab washing was inoculated via the allontoic route of 10-d-old specific-pathogen-free chicken embryos (3 embryos per swab washing), followed by incubation of the inoculated embryos for three days at 37.8^o^ C and 60% relative humidity. Allantoic fluids were collected from the incubated embryos, and their individual HA titers were determined against chicken red blood cell suspension of 0.5% [66]. HA titers that were greater ≥ 1:40 were considered positive for H9N2 recovery from the chicken trachea.

#### Protection against tracheal histopathologic lesions

The histopathology work included the collection of tracheas from 20 randomly selected birds of each of the six TRTs at 35 d of age, and preserving the tracheas in 10% formalin, before subjecting them to H and E staining [67, 68]Three circular sections, each of 5 μm thickness were collected from each formalized trachea at cranial, middle, and caudal positions. The microscopic lesions were recorded in each circular section at 12, 15, 30, and 45 min-positions of an analog watch, under a microscope magnification of 400X. The recorded lesions included deciliation, mucosal hypertrophy, and goblet cells degeneration; images of these tracheal lesions were documented previously [52]. The mean frequency of each lesion was compared statistically among the birds of the six treatments.

### Production performance

The cumulative mortality, live body weight, and feed conversion ratio (FCR) (consumed feed divided by live body weight) in each replicate pen of the six treatments were recorded and their means per treatment were calculated.

### Statistical analysis

The mean ELISA O.D. values obtained for the IgA and IgG of the tracheobronchial washings that are specific to H9N2 and those specific to MG, and the means of live body weight and FCR were compared among the six treatments by ANOVA for completely randomized design; however, the means of the MG count, frequency of tracheal H9N2 recovery, frequency of tracheal histopathologic lesions, and mortality % were compared between treatments using the CHI Square (MSTAT, version 6.5.1, Michigan State University, MI, USA).

## Data Availability

The datasets and analysis are available upon requests made to the E-mail address of the manuscript’s corresponding author.

## Abbreviations

ANOVA: Analysis of variance
CRD: Completely randomized design
d: Day
EE: *Echinacea* extract
ELISA: Enzyme-linked immunosorbent assay
IgA: Immunoglobulin A
IgG: Immunoglobulin G
Iu: International units
MENA: Middle East and North Africa
MG: *Mycoplasma gallisepticum*
μg: Microgram
μL: Microliter
μm: Micrometer
mg: Milligram
nm: Nanometer
OIE: Office Internationale des Epizooties
TRT: Treatment
TRTS: Treatments
